# 3-[4-(2-Amino-2-oxoeth­yl)phen­oxy]-2-hy­droxy-*N*-isopropyl­propanaminium 1,1′-binaphthyl-2,2′-diyl phosphate

**DOI:** 10.1107/S1600536810049494

**Published:** 2010-12-11

**Authors:** En-Ju Wang, Guang-Ying Chen

**Affiliations:** a Hainan Provincial Key Laboratory of Tropical Pharmaceutical Herb Chemistry, School of Chemistry and Chemical Engineering, Hainan Normal University, Haikou 571158, People’s Republic of China

## Abstract

In the title salt, C_14_H_23_N_2_O_3_^+^·C_20_H_12_O_4_P^−^, the dihedral angle between the two naphthyl ring systems in the anion is 57.77 (6)°. In the crystal, an O—H⋯O hydrogen bond links the components. The ammonium group engages in N—H⋯O hydrogen bonds, generating a layer structure.

## Related literature

For the uses of BINOL-phospho­ric acid, see: Jacques *et al.* (1971 ▶); Sewgobind *et al.*, (2008 ▶). For a clinical pharmacological study of atenolol [systematic name: 3-(4-(2-amino-2-oxoeth­yl)phen­oxy)-2-hy­droxy-*N*-isopropyl­propan-1-amine], see: Agon *et al.* (1991 ▶). For the stereoselective features of atenolol, see: Stoschitzky *et al.* (1993 ▶). 
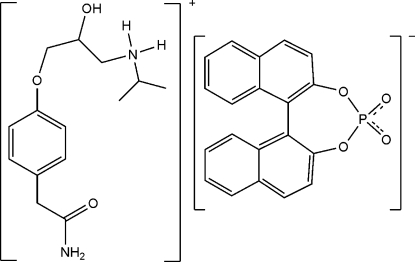


## Experimental

### 

#### Crystal data

C_14_H_23_N_2_O_3_^+^·C_20_H_12_O_4_P^−^
*M* *_r_* = 614.61
Orthorhombic, 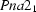

*a* = 9.8646 (14) Å
*b* = 26.145 (4) Å
*c* = 11.9306 (16) Å
*V* = 3077.1 (7) Å^3^
*Z* = 4
Mo *K*α radiation
μ = 0.14 mm^−1^
*T* = 273 K
0.39 × 0.38 × 0.35 mm

#### Data collection

Bruker SMART diffractometer
Absorption correction: multi-scan (*SADABS*; Sheldrick, 1996 ▶) *T*_min_ = 0.947, *T*_max_ = 0.952
14697 measured reflections
5261 independent reflections
3982 reflections with *I* > 2σ(*I*)
*R*_int_ = 0.035

#### Refinement

*R*[*F* ^2^ > 2σ(*F* ^2^)] = 0.038
*wR*(*F* ^2^) = 0.088
*S* = 1.06
5261 reflections
421 parameters
1 restraint
H-atom parameters constrained
Δρ_max_ = 0.28 e Å^−3^
Δρ_min_ = −0.22 e Å^−3^
Absolute structure: Flack (1983 ▶), 2855 Friedel pairs
Flack parameter: 0.03 (10)

### 

Data collection: *SMART* (Bruker, 2001 ▶); cell refinement: *SAINT* (Bruker, 2001 ▶); data reduction: *SAINT*; program(s) used to solve structure: *SHELXS97* (Sheldrick, 2008 ▶); program(s) used to refine structure: *SHELXL97* (Sheldrick, 2008 ▶); molecular graphics: *SHELXTL* (Sheldrick, 2008 ▶); software used to prepare material for publication: *SHELXTL*.

## Supplementary Material

Crystal structure: contains datablocks I, global. DOI: 10.1107/S1600536810049494/ng5070sup1.cif

Structure factors: contains datablocks I. DOI: 10.1107/S1600536810049494/ng5070Isup2.hkl

Additional supplementary materials:  crystallographic information; 3D view; checkCIF report

## Figures and Tables

**Table 1:** Hydrogen-bond geometry (Å, °)

*D*—H⋯*A*	*D*—H	H⋯*A*	*D*⋯*A*	*D*—H⋯*A*
O7—H7*A*⋯O3	0.82	1.87	2.683 (3)	172
N2—H2*B*⋯O4^i^	0.90	1.95	2.746 (3)	147
N2—H2*A*⋯O5^ii^	0.90	1.96	2.831 (3)	163
N1—H1*B*⋯O5^i^	0.86	2.17	2.960 (3)	153
N1—H1*A*⋯O4^iii^	0.86	2.07	2.907 (3)	164

Symmetry codes: (i) 
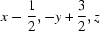
; (ii) 
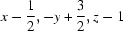
; (iii) 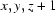.

**Fig. 1.**
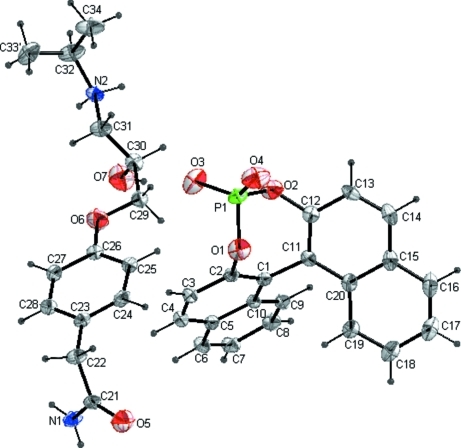
Molecular configuration and atom numbering scheme for the title compound (I), with displacement ellipsoids drawn at the 30% probability level.

**Fig. 2.**
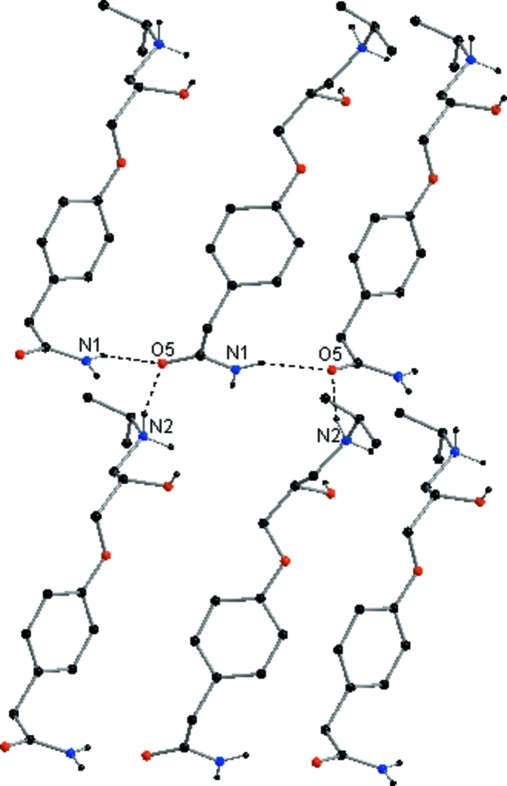
The partial packing diagram of (I), showing the molecular layer of atenolol. Hydrogen bonds are shown as dashed lines. H atoms bonded to C atoms have been omitted for clarity.

**Fig. 3.**
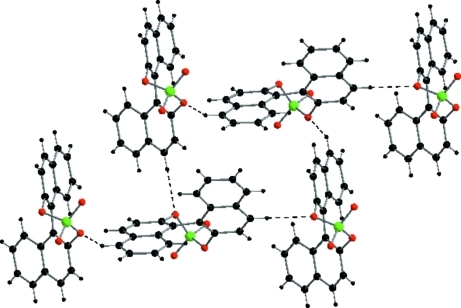
The partial packing of diagram(I), showing the molecular layer of BINOL-phosphoric acid linked via C—H···O interactions.

**Fig. 4.**
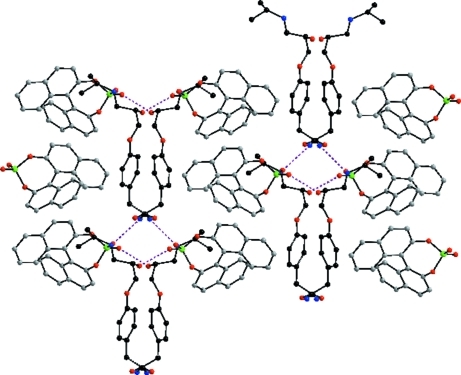
The partial packing of diagram(I), showing interactions between molecular layers. H atoms have been omitted for clarity.

## supplementary crystallographic information

### Comment

The title compound is a salt of BINOL-phosphoric acid. It represents a useful tool for the resolution of chiral amines (Jacques *et al.*, 1971). BINOL-phosphoric acid has also been used to catalyze enantioselective Pictet-Spengler reactions in good yields and excellent ee values. (Sewgobind *et al.*, 2008).

3-(4-(2-Amino-2-oxoethyl)phenoxy)-2-hydroxy-*N*-isopropylpropan-1-amine, known as atenolol, is a selective β_1_ receptor antagonist that belongs to the group of β-blockers, a class of drugs used primarily in cardiovascular diseases (Agon *et al.*, 1991). It is a chiral drug. Only (S)-atenolol contributes to the β-blocking effect (Stoschitzky *et al.*, 1993).

In the title salt, (C_14_H_23_N_2_O_3_)^+^.(C_20_H_12_O_4_P)^-^(I) (Figure 1), the (C_14_H_23_N_2_O_3_)^+ ^and the (C_20_H_12_O_4_P)^- ^are separately linked into two molecular layers (Figure 2 and figure 3). The two types of layers are alternately stacked to give a layered network. An N—H···O interaction links the cation to the anion that contributes to the interaction between the adjacent molecular layers. There are other two hydrogen bonding interactions to link the layers together (Figure 4).

### Experimental

A mixture of BINOL-phosphoric acid (1 mmoL, 0.35 g) and atenolol (1 mmol,0.27 g) in 10 mL ethanol was heat to 60 °C and stirred. After the suspended solid dissolved, the solution was stirred for another 2 h for the salifying process to be fully completed. The filtered solution was kept at 20 °C. Colorless crystals suitable for the single X-ray diffraction were obtained in one week.

### Refinement

H atoms bonded to C were positioned geometrically with aromatic C—H = 0.93 Å and aliphatic C—H = 0.96–0.98 Å. Their displacement parameters were set for methyl H atoms at *U*_iso_(H) = 1.5*U*_eq_(C) and for aromatic and other aliphatic H atoms at *U*_iso_(H)= 1.2*U*_eq_(C). The hydroxyl H atom and the amino H atoms were found in Fourier difference maps and refined with the constraints of N—H = 0.86–0.90 Å, *U*_iso_(H)= 1.2*U*_eq_(N) and O—H = 0.82 Å, *U*_iso_(H) = 1.2*U*_eq_(N).

### Crystal data

**Table:**

C_14_H_23_N_2_O_3_^+^·C_20_H_12_O_4_P^−^	*D*_x_ = 1.327 Mg m^−^^3^
*M**_r_* = 614.61	Mo *K*α radiation, λ = 0.71073 Å
Orthorhombic, *P**n**a*2_1_	Cell parameters from 4710 reflections
*a* = 9.8646 (14) Å	θ = 2.6–22.7°
*b* = 26.145 (4) Å	µ = 0.14 mm^−^^1^
*c* = 11.9306 (16) Å	*T* = 273 K
*V* = 3077.1 (7) Å^3^	Plate, colorless
*Z* = 4	0.39 × 0.38 × 0.35 mm
*F*(000) = 1296	

### Data collection

**Table:**

Bruker SMART diffractometer	5261 independent reflections
Radiation source: fine-focus sealed tube	3982 reflections with *I* > 2σ(*I*)
graphite	*R*_int_ = 0.035
phi and ω scans	θ_max_ = 25.0°, θ_min_ = 1.6°
Absorption correction: multi-scan (*SADABS*; Sheldrick, 1996)	*h* = −11→11
*T*_min_ = 0.947, *T*_max_ = 0.952	*k* = −19→31
14697 measured reflections	*l* = −14→14

### Refinement

**Table:**

Refinement on *F*^2^	Secondary atom site location: difference Fourier map
Least-squares matrix: full	Hydrogen site location: inferred from neighbouring sites
*R*[*F*^2^ > 2σ(*F*^2^)] = 0.038	H-atom parameters constrained
*wR*(*F*^2^) = 0.088	*w* = 1/[σ^2^(*F*_o_^2^) + (0.0409*P*)^2^ + 0.2334*P*] where *P* = (*F*_o_^2^ + 2*F*_c_^2^)/3
*S* = 1.06	(Δ/σ)_max_ = 0.001
5261 reflections	Δρ_max_ = 0.28 e Å^−^^3^
421 parameters	Δρ_min_ = −0.22 e Å^−^^3^
1 restraint	Absolute structure: Flack (1983), 2855 Friedel pairs
Primary atom site location: structure-invariant direct methods	Flack parameter: 0.03 (10)

### Special details

**Table:**

Geometry. All e.s.d.'s (except the e.s.d. in the dihedral angle between two l.s. planes) are estimated using the full covariance matrix. The cell e.s.d.'s are taken into account individually in the estimation of e.s.d.'s in distances, angles and torsion angles; correlations between e.s.d.'s in cell parameters are only used when they are defined by crystal symmetry. An approximate (isotropic) treatment of cell e.s.d.'s is used for estimating e.s.d.'s involving l.s. planes.
Refinement. Refinement of *F*^2^ against ALL reflections. The weighted *R*-factor *wR* and goodness of fit *S* are based on *F*^2^, conventional *R*-factors *R* are based on *F*, with *F* set to zero for negative *F*^2^. The threshold expression of *F*^2^ > σ(*F*^2^) is used only for calculating *R*-factors(gt) *etc*. and is not relevant to the choice of reflections for refinement. *R*-factors based on *F*^2^ are statistically about twice as large as those based on *F*, and *R*- factors based on ALL data will be even larger.

### Fractional atomic coordinates and isotropic or equivalent isotropic displacement parameters (Å^2^)

**Table:**

	*x*	*y*	*z*	*U*_iso_*/*U*_eq_	Occ. (<1)
N1	1.0356 (2)	0.73553 (9)	0.9249 (2)	0.0503 (6)	
H1A	1.0214	0.7081	0.9628	0.060*	
H1B	0.9688	0.7550	0.9070	0.060*	
N2	0.7574 (2)	0.84866 (8)	0.09975 (18)	0.0425 (6)	
H2A	0.7757	0.8256	0.0458	0.051*	
H2B	0.6727	0.8425	0.1243	0.051*	
O1	0.85501 (19)	0.60766 (7)	0.23253 (15)	0.0414 (5)	
O2	0.79646 (19)	0.59375 (7)	0.03175 (14)	0.0428 (5)	
O3	0.72831 (19)	0.67462 (7)	0.11765 (19)	0.0537 (5)	
O4	0.97968 (19)	0.65639 (8)	0.08968 (18)	0.0565 (6)	
O5	1.2588 (2)	0.72090 (7)	0.91795 (17)	0.0487 (5)	
O6	0.9002 (2)	0.79899 (8)	0.41140 (16)	0.0508 (5)	
O7	0.7363 (2)	0.76326 (8)	0.2308 (2)	0.0639 (6)	
H7A	0.7331	0.7348	0.2019	0.096*	
P1	0.84174 (7)	0.63871 (3)	0.11563 (7)	0.04164 (19)	
C1	0.7172 (3)	0.53332 (10)	0.2126 (2)	0.0354 (6)	
C2	0.7427 (3)	0.57925 (10)	0.2645 (2)	0.0363 (6)	
C3	0.6600 (3)	0.59822 (11)	0.3496 (2)	0.0427 (7)	
H3	0.6821	0.6288	0.3849	0.051*	
C4	0.5485 (3)	0.57245 (11)	0.3810 (3)	0.0468 (7)	
H4	0.4960	0.5849	0.4397	0.056*	
C5	0.5094 (3)	0.52674 (10)	0.3266 (2)	0.0396 (7)	
C6	0.3886 (3)	0.50032 (12)	0.3525 (3)	0.0515 (8)	
H6	0.3353	0.5116	0.4118	0.062*	
C7	0.3484 (3)	0.45894 (12)	0.2931 (3)	0.0532 (8)	
H7	0.2684	0.4422	0.3118	0.064*	
C8	0.4272 (3)	0.44137 (11)	0.2032 (3)	0.0499 (8)	
H8	0.3978	0.4137	0.1606	0.060*	
C9	0.5465 (3)	0.46459 (11)	0.1783 (2)	0.0425 (7)	
H9	0.5986	0.4519	0.1196	0.051*	
C10	0.5938 (3)	0.50747 (10)	0.2389 (2)	0.0349 (6)	
C11	0.8142 (3)	0.51381 (9)	0.1265 (2)	0.0352 (6)	
C12	0.8525 (3)	0.54511 (10)	0.0395 (2)	0.0384 (6)	
C13	0.9387 (3)	0.52908 (12)	−0.0454 (2)	0.0464 (8)	
H13	0.9626	0.5515	−0.1026	0.056*	
C14	0.9877 (3)	0.48104 (13)	−0.0448 (3)	0.0568 (9)	
H14	1.0420	0.4701	−0.1038	0.068*	
C15	0.9584 (3)	0.44693 (11)	0.0438 (3)	0.0501 (8)	
C16	1.0124 (4)	0.39660 (14)	0.0491 (4)	0.0739 (11)	
H16	1.0673	0.3851	−0.0090	0.089*	
C17	0.9863 (4)	0.36521 (13)	0.1357 (4)	0.0715 (11)	
H17	1.0225	0.3324	0.1366	0.086*	
C18	0.9060 (4)	0.38159 (12)	0.2232 (3)	0.0611 (9)	
H18	0.8897	0.3599	0.2835	0.073*	
C19	0.8500 (3)	0.42961 (11)	0.2220 (3)	0.0479 (8)	
H19	0.7961	0.4400	0.2817	0.058*	
C20	0.8726 (3)	0.46360 (10)	0.1314 (2)	0.0396 (7)	
C21	1.1587 (3)	0.74762 (11)	0.8944 (2)	0.0396 (7)	
C22	1.1765 (3)	0.79671 (11)	0.8281 (3)	0.0499 (8)	
H22A	1.1437	0.8251	0.8729	0.060*	
H22B	1.2724	0.8021	0.8148	0.060*	
C23	1.1032 (3)	0.79705 (10)	0.7164 (2)	0.0419 (7)	
C24	1.1619 (3)	0.77574 (10)	0.6244 (3)	0.0510 (7)	
H24	1.2475	0.7612	0.6319	0.061*	
C25	1.1003 (3)	0.77459 (11)	0.5198 (3)	0.0499 (8)	
H25	1.1425	0.7593	0.4587	0.060*	
C26	0.9733 (3)	0.79712 (10)	0.5096 (2)	0.0414 (7)	
C27	0.9134 (3)	0.81967 (10)	0.6010 (2)	0.0442 (7)	
H27	0.8295	0.8355	0.5936	0.053*	
C28	0.9773 (3)	0.81891 (11)	0.7032 (2)	0.0460 (7)	
H28	0.9345	0.8335	0.7649	0.055*	
C29	0.9557 (3)	0.77359 (12)	0.3161 (2)	0.0495 (8)	
H29A	0.9613	0.7371	0.3302	0.059*	
H29B	1.0463	0.7863	0.3011	0.059*	
C30	0.8654 (3)	0.78359 (11)	0.2172 (3)	0.0476 (8)	
H30	0.9066	0.7675	0.1513	0.057*	
C31	0.8528 (3)	0.83978 (11)	0.1944 (3)	0.0524 (8)	
H31A	0.8197	0.8571	0.2610	0.063*	
H31B	0.9411	0.8538	0.1760	0.063*	
C32	0.7597 (4)	0.90039 (13)	0.0483 (3)	0.0705 (11)	
H32	0.6919	0.9007	−0.0117	0.085*	
C33	0.702 (3)	0.9354 (9)	0.150 (2)	0.071 (5)	0.52 (5)
H33A	0.6246	0.9189	0.1829	0.106*	0.52 (5)
H33B	0.6755	0.9682	0.1211	0.106*	0.52 (5)
H33C	0.7711	0.9397	0.2055	0.106*	0.52 (5)
C34	0.885 (4)	0.9195 (11)	0.002 (3)	0.084 (6)	0.52 (5)
H34A	0.9543	0.9183	0.0582	0.127*	0.52 (5)
H34B	0.8723	0.9542	−0.0222	0.127*	0.52 (5)
H34C	0.9109	0.8987	−0.0608	0.127*	0.52 (5)
C33'	0.736 (3)	0.9449 (9)	0.112 (3)	0.071 (5)	0.48 (5)
H33D	0.6466	0.9438	0.1426	0.106*	0.48 (5)
H33E	0.7454	0.9744	0.0644	0.106*	0.48 (5)
H33F	0.8015	0.9468	0.1714	0.106*	0.48 (5)
C34'	0.905 (4)	0.9000 (11)	−0.014 (3)	0.084 (6)	0.48 (5)
H34D	0.9755	0.9062	0.0400	0.127*	0.48 (5)
H34E	0.9069	0.9262	−0.0702	0.127*	0.48 (5)
H34F	0.9193	0.8672	−0.0485	0.127*	0.48 (5)

### Atomic displacement parameters (Å^2^)

**Table:**

	*U*^11^	*U*^22^	*U*^33^	*U*^12^	*U*^13^	*U*^23^
N1	0.0371 (16)	0.0527 (16)	0.0611 (16)	0.0033 (12)	−0.0007 (13)	0.0174 (13)
N2	0.0456 (14)	0.0460 (13)	0.0359 (13)	−0.0060 (10)	−0.0034 (11)	−0.0012 (11)
O1	0.0418 (12)	0.0404 (11)	0.0420 (10)	−0.0062 (9)	−0.0069 (9)	−0.0006 (9)
O2	0.0485 (12)	0.0423 (11)	0.0376 (11)	−0.0027 (9)	−0.0046 (9)	0.0038 (9)
O3	0.0509 (12)	0.0416 (11)	0.0686 (13)	0.0055 (9)	−0.0095 (12)	0.0013 (11)
O4	0.0455 (13)	0.0587 (13)	0.0654 (14)	−0.0107 (10)	−0.0040 (11)	0.0149 (11)
O5	0.0364 (12)	0.0554 (12)	0.0542 (12)	0.0032 (10)	0.0005 (10)	0.0106 (10)
O6	0.0526 (13)	0.0634 (13)	0.0364 (11)	0.0136 (10)	−0.0030 (10)	0.0002 (10)
O7	0.0598 (16)	0.0606 (15)	0.0712 (16)	−0.0103 (12)	0.0109 (12)	−0.0049 (12)
P1	0.0403 (4)	0.0378 (4)	0.0469 (4)	−0.0062 (3)	−0.0050 (4)	0.0061 (4)
C1	0.0380 (17)	0.0374 (16)	0.0307 (14)	0.0030 (13)	−0.0012 (12)	0.0042 (12)
C2	0.0399 (17)	0.0367 (16)	0.0323 (15)	0.0015 (14)	−0.0047 (13)	0.0032 (12)
C3	0.053 (2)	0.0409 (16)	0.0345 (15)	0.0052 (15)	−0.0024 (15)	−0.0028 (13)
C4	0.055 (2)	0.0487 (18)	0.0364 (15)	0.0123 (16)	0.0069 (14)	0.0003 (14)
C5	0.0403 (18)	0.0439 (16)	0.0348 (15)	0.0077 (14)	0.0027 (13)	0.0064 (14)
C6	0.047 (2)	0.059 (2)	0.0478 (18)	0.0068 (16)	0.0154 (15)	0.0117 (16)
C7	0.046 (2)	0.053 (2)	0.061 (2)	−0.0061 (16)	0.0108 (17)	0.0140 (17)
C8	0.050 (2)	0.0425 (18)	0.0577 (19)	−0.0066 (14)	0.0051 (17)	0.0035 (15)
C9	0.0440 (19)	0.0417 (17)	0.0418 (16)	0.0002 (14)	0.0054 (14)	−0.0025 (13)
C10	0.0350 (16)	0.0351 (15)	0.0347 (14)	0.0017 (13)	0.0036 (13)	0.0068 (12)
C11	0.0339 (15)	0.0376 (14)	0.0340 (14)	−0.0049 (11)	−0.0004 (13)	−0.0043 (13)
C12	0.0338 (16)	0.0404 (16)	0.0410 (16)	−0.0062 (13)	−0.0027 (13)	−0.0013 (13)
C13	0.0397 (18)	0.059 (2)	0.0403 (17)	−0.0098 (15)	0.0072 (14)	0.0009 (15)
C14	0.045 (2)	0.069 (2)	0.055 (2)	0.0013 (17)	0.0150 (16)	−0.0134 (19)
C15	0.0414 (19)	0.0492 (19)	0.060 (2)	0.0005 (14)	0.0068 (16)	−0.0107 (16)
C16	0.067 (3)	0.058 (2)	0.096 (3)	0.0145 (19)	0.019 (2)	−0.020 (2)
C17	0.071 (3)	0.045 (2)	0.099 (3)	0.0164 (18)	0.001 (2)	−0.006 (2)
C18	0.064 (2)	0.0431 (19)	0.076 (2)	0.0054 (17)	−0.010 (2)	−0.0019 (18)
C19	0.049 (2)	0.0415 (17)	0.0533 (19)	0.0032 (15)	−0.0030 (15)	−0.0024 (15)
C20	0.0361 (17)	0.0376 (16)	0.0450 (16)	−0.0025 (12)	−0.0051 (13)	−0.0069 (15)
C21	0.0391 (18)	0.0433 (17)	0.0363 (15)	−0.0004 (14)	−0.0080 (14)	0.0009 (13)
C22	0.055 (2)	0.0433 (17)	0.0517 (18)	−0.0073 (15)	−0.0076 (15)	0.0095 (15)
C23	0.0463 (19)	0.0354 (15)	0.0441 (18)	−0.0014 (14)	−0.0002 (15)	0.0104 (13)
C24	0.0445 (17)	0.0519 (18)	0.0565 (19)	0.0140 (14)	−0.0032 (17)	0.0130 (18)
C25	0.053 (2)	0.0537 (19)	0.0425 (17)	0.0178 (16)	0.0052 (16)	0.0058 (15)
C26	0.0480 (19)	0.0389 (17)	0.0374 (16)	0.0036 (14)	0.0005 (14)	0.0060 (13)
C27	0.0429 (16)	0.0456 (17)	0.0442 (17)	0.0067 (12)	0.0010 (16)	0.0032 (15)
C28	0.053 (2)	0.0443 (18)	0.0410 (17)	−0.0002 (15)	0.0060 (15)	0.0037 (14)
C29	0.051 (2)	0.0525 (18)	0.0446 (18)	0.0074 (16)	0.0003 (15)	−0.0046 (15)
C30	0.046 (2)	0.0487 (18)	0.0479 (18)	−0.0063 (14)	0.0026 (15)	−0.0040 (15)
C31	0.060 (2)	0.0522 (19)	0.0454 (18)	−0.0136 (16)	−0.0161 (16)	0.0047 (15)
C32	0.080 (3)	0.063 (2)	0.068 (2)	−0.017 (2)	−0.036 (2)	0.013 (2)
C33	0.081 (12)	0.046 (8)	0.085 (13)	−0.006 (6)	−0.030 (9)	−0.003 (7)
C34	0.107 (13)	0.069 (12)	0.077 (10)	−0.037 (13)	−0.007 (9)	0.022 (12)
C33'	0.081 (13)	0.046 (9)	0.085 (14)	−0.006 (6)	−0.030 (10)	−0.003 (7)
C34'	0.107 (13)	0.069 (13)	0.077 (11)	−0.037 (14)	−0.007 (9)	0.022 (12)

### Geometric parameters (Å, °)

**Table:**

N1—C21	1.306 (3)	C16—H16	0.9300
N1—H1A	0.8600	C17—C18	1.378 (5)
N1—H1B	0.8600	C17—H17	0.9300
N2—C32	1.485 (4)	C18—C19	1.372 (4)
N2—C31	1.488 (4)	C18—H18	0.9300
N2—H2A	0.9000	C19—C20	1.417 (4)
N2—H2B	0.9000	C19—H19	0.9300
O1—C2	1.388 (3)	C21—C22	1.518 (4)
O1—P1	1.6189 (19)	C22—C23	1.516 (4)
O2—C12	1.390 (3)	C22—H22A	0.9700
O2—P1	1.6071 (19)	C22—H22B	0.9700
O3—P1	1.4608 (19)	C23—C24	1.361 (4)
O4—P1	1.470 (2)	C23—C28	1.377 (4)
O5—C21	1.242 (3)	C24—C25	1.388 (4)
O6—C26	1.376 (3)	C24—H24	0.9300
O6—C29	1.426 (3)	C25—C26	1.390 (4)
O7—C30	1.389 (3)	C25—H25	0.9300
O7—H7A	0.8200	C26—C27	1.373 (4)
C1—C2	1.374 (4)	C27—C28	1.373 (4)
C1—C10	1.428 (4)	C27—H27	0.9300
C1—C11	1.494 (4)	C28—H28	0.9300
C2—C3	1.394 (4)	C29—C30	1.502 (4)
C3—C4	1.343 (4)	C29—H29A	0.9700
C3—H3	0.9300	C29—H29B	0.9700
C4—C5	1.414 (4)	C30—C31	1.499 (4)
C4—H4	0.9300	C30—H30	0.9800
C5—C6	1.412 (4)	C31—H31A	0.9700
C5—C10	1.428 (4)	C31—H31B	0.9700
C6—C7	1.353 (4)	C32—C33'	1.41 (3)
C6—H6	0.9300	C32—C34	1.44 (3)
C7—C8	1.402 (4)	C32—C34'	1.62 (4)
C7—H7	0.9300	C32—C33	1.62 (3)
C8—C9	1.356 (4)	C32—H32	0.9800
C8—H8	0.9300	C33—H33A	0.9600
C9—C10	1.413 (4)	C33—H33B	0.9600
C9—H9	0.9300	C33—H33C	0.9600
C11—C12	1.374 (4)	C34—H34A	0.9600
C11—C20	1.435 (3)	C34—H34B	0.9600
C12—C13	1.388 (4)	C34—H34C	0.9600
C13—C14	1.346 (4)	C33'—H33D	0.9600
C13—H13	0.9300	C33'—H33E	0.9600
C14—C15	1.412 (4)	C33'—H33F	0.9600
C14—H14	0.9300	C34'—H34D	0.9600
C15—C20	1.413 (4)	C34'—H34E	0.9600
C15—C16	1.421 (5)	C34'—H34F	0.9600
C16—C17	1.344 (5)		
			
C21—N1—H1A	120.0	O5—C21—N1	122.7 (3)
C21—N1—H1B	120.0	O5—C21—C22	120.1 (3)
H1A—N1—H1B	120.0	N1—C21—C22	117.2 (3)
C32—N2—C31	116.5 (2)	C23—C22—C21	114.1 (2)
C32—N2—H2A	108.2	C23—C22—H22A	108.7
C31—N2—H2A	108.2	C21—C22—H22A	108.7
C32—N2—H2B	108.2	C23—C22—H22B	108.7
C31—N2—H2B	108.2	C21—C22—H22B	108.7
H2A—N2—H2B	107.3	H22A—C22—H22B	107.6
C2—O1—P1	116.13 (16)	C24—C23—C28	117.5 (3)
C12—O2—P1	121.14 (16)	C24—C23—C22	120.3 (3)
C26—O6—C29	117.5 (2)	C28—C23—C22	122.2 (3)
C30—O7—H7A	109.5	C23—C24—C25	123.2 (3)
O3—P1—O4	120.69 (12)	C23—C24—H24	118.4
O3—P1—O2	105.51 (11)	C25—C24—H24	118.4
O4—P1—O2	110.86 (12)	C24—C25—C26	117.7 (3)
O3—P1—O1	111.71 (12)	C24—C25—H25	121.1
O4—P1—O1	105.32 (11)	C26—C25—H25	121.1
O2—P1—O1	101.09 (9)	C27—C26—O6	115.8 (2)
C2—C1—C10	118.1 (2)	C27—C26—C25	120.0 (3)
C2—C1—C11	119.4 (2)	O6—C26—C25	124.2 (3)
C10—C1—C11	122.4 (2)	C28—C27—C26	120.2 (3)
C1—C2—O1	119.3 (2)	C28—C27—H27	119.9
C1—C2—C3	122.2 (3)	C26—C27—H27	119.9
O1—C2—C3	118.5 (2)	C27—C28—C23	121.4 (3)
C4—C3—C2	120.2 (3)	C27—C28—H28	119.3
C4—C3—H3	119.9	C23—C28—H28	119.3
C2—C3—H3	119.9	O6—C29—C30	108.5 (2)
C3—C4—C5	121.3 (3)	O6—C29—H29A	110.0
C3—C4—H4	119.4	C30—C29—H29A	110.0
C5—C4—H4	119.4	O6—C29—H29B	110.0
C6—C5—C4	122.9 (3)	C30—C29—H29B	110.0
C6—C5—C10	118.6 (3)	H29A—C29—H29B	108.4
C4—C5—C10	118.4 (3)	O7—C30—C31	108.7 (2)
C7—C6—C5	121.6 (3)	O7—C30—C29	112.7 (3)
C7—C6—H6	119.2	C31—C30—C29	111.2 (2)
C5—C6—H6	119.2	O7—C30—H30	108.0
C6—C7—C8	120.0 (3)	C31—C30—H30	108.0
C6—C7—H7	120.0	C29—C30—H30	108.0
C8—C7—H7	120.0	N2—C31—C30	110.0 (2)
C9—C8—C7	120.1 (3)	N2—C31—H31A	109.7
C9—C8—H8	119.9	C30—C31—H31A	109.7
C7—C8—H8	119.9	N2—C31—H31B	109.7
C8—C9—C10	121.9 (3)	C30—C31—H31B	109.7
C8—C9—H9	119.0	H31A—C31—H31B	108.2
C10—C9—H9	119.0	C33'—C32—C34	93.4 (11)
C9—C10—C1	123.0 (2)	C33'—C32—N2	121.9 (13)
C9—C10—C5	117.5 (3)	C34—C32—N2	119.1 (13)
C1—C10—C5	119.4 (2)	C33'—C32—C34'	113.4 (12)
C12—C11—C20	117.7 (2)	C34—C32—C34'	20.9 (11)
C12—C11—C1	119.5 (2)	N2—C32—C34'	101.3 (12)
C20—C11—C1	122.8 (2)	C33'—C32—C33	22.2 (9)
C11—C12—C13	122.7 (3)	C34—C32—C33	113.1 (12)
C11—C12—O2	119.1 (2)	N2—C32—C33	101.6 (10)
C13—C12—O2	118.1 (2)	C34'—C32—C33	131.3 (12)
C14—C13—C12	119.9 (3)	C33'—C32—H32	105.9
C14—C13—H13	120.1	C34—C32—H32	107.5
C12—C13—H13	120.1	N2—C32—H32	107.5
C13—C14—C15	121.3 (3)	C34'—C32—H32	105.7
C13—C14—H14	119.3	C33—C32—H32	107.5
C15—C14—H14	119.3	C32—C33—H33A	109.5
C14—C15—C20	118.8 (3)	C32—C33—H33B	109.5
C14—C15—C16	122.7 (3)	C32—C33—H33C	109.5
C20—C15—C16	118.5 (3)	C32—C34—H34A	109.5
C17—C16—C15	121.9 (3)	C32—C34—H34B	109.5
C17—C16—H16	119.1	C32—C34—H34C	109.5
C15—C16—H16	119.1	C32—C33'—H33D	109.5
C16—C17—C18	120.1 (3)	C32—C33'—H33E	109.5
C16—C17—H17	119.9	H33D—C33'—H33E	109.5
C18—C17—H17	119.9	C32—C33'—H33F	109.5
C19—C18—C17	120.5 (3)	H33D—C33'—H33F	109.5
C19—C18—H18	119.7	H33E—C33'—H33F	109.5
C17—C18—H18	119.7	C32—C34'—H34D	109.5
C18—C19—C20	121.2 (3)	C32—C34'—H34E	109.5
C18—C19—H19	119.4	H34D—C34'—H34E	109.5
C20—C19—H19	119.4	C32—C34'—H34F	109.5
C15—C20—C19	117.7 (2)	H34D—C34'—H34F	109.5
C15—C20—C11	119.5 (3)	H34E—C34'—H34F	109.5
C19—C20—C11	122.8 (3)		
			
C12—O2—P1—O3	−155.99 (19)	C12—C13—C14—C15	−3.0 (4)
C12—O2—P1—O4	71.7 (2)	C13—C14—C15—C20	1.8 (4)
C12—O2—P1—O1	−39.5 (2)	C13—C14—C15—C16	−177.7 (3)
C2—O1—P1—O3	58.6 (2)	C14—C15—C16—C17	178.1 (3)
C2—O1—P1—O4	−168.71 (18)	C20—C15—C16—C17	−1.4 (5)
C2—O1—P1—O2	−53.25 (19)	C15—C16—C17—C18	−0.5 (6)
C10—C1—C2—O1	−173.7 (2)	C16—C17—C18—C19	1.2 (5)
C11—C1—C2—O1	2.7 (4)	C17—C18—C19—C20	0.1 (5)
C10—C1—C2—C3	7.1 (4)	C14—C15—C20—C19	−177.0 (3)
C11—C1—C2—C3	−176.5 (2)	C16—C15—C20—C19	2.5 (4)
P1—O1—C2—C1	76.7 (3)	C14—C15—C20—C11	2.1 (4)
P1—O1—C2—C3	−104.1 (2)	C16—C15—C20—C11	−178.5 (3)
C1—C2—C3—C4	−2.4 (4)	C18—C19—C20—C15	−1.9 (4)
O1—C2—C3—C4	178.4 (2)	C18—C19—C20—C11	179.1 (3)
C2—C3—C4—C5	−2.2 (4)	C12—C11—C20—C15	−4.5 (4)
C3—C4—C5—C6	−175.9 (3)	C1—C11—C20—C15	176.8 (2)
C3—C4—C5—C10	1.9 (4)	C12—C11—C20—C19	174.5 (3)
C4—C5—C6—C7	174.7 (3)	C1—C11—C20—C19	−4.2 (4)
C10—C5—C6—C7	−3.1 (4)	O5—C21—C22—C23	118.5 (3)
C5—C6—C7—C8	−0.1 (5)	N1—C21—C22—C23	−61.8 (4)
C6—C7—C8—C9	2.5 (5)	C21—C22—C23—C24	−84.0 (4)
C7—C8—C9—C10	−1.6 (4)	C21—C22—C23—C28	96.6 (3)
C8—C9—C10—C1	−178.4 (3)	C28—C23—C24—C25	−0.9 (4)
C8—C9—C10—C5	−1.6 (4)	C22—C23—C24—C25	179.8 (3)
C2—C1—C10—C9	169.6 (2)	C23—C24—C25—C26	1.0 (5)
C11—C1—C10—C9	−6.7 (4)	C29—O6—C26—C27	−176.7 (3)
C2—C1—C10—C5	−7.2 (4)	C29—O6—C26—C25	3.7 (4)
C11—C1—C10—C5	176.5 (2)	C24—C25—C26—C27	0.3 (4)
C6—C5—C10—C9	3.9 (4)	C24—C25—C26—O6	179.9 (3)
C4—C5—C10—C9	−174.0 (3)	O6—C26—C27—C28	178.7 (2)
C6—C5—C10—C1	−179.2 (2)	C25—C26—C27—C28	−1.6 (4)
C4—C5—C10—C1	2.9 (4)	C26—C27—C28—C23	1.8 (4)
C2—C1—C11—C12	−51.5 (3)	C24—C23—C28—C27	−0.5 (4)
C10—C1—C11—C12	124.7 (3)	C22—C23—C28—C27	178.8 (3)
C2—C1—C11—C20	127.1 (3)	C26—O6—C29—C30	−176.0 (2)
C10—C1—C11—C20	−56.7 (4)	O6—C29—C30—O7	−63.6 (3)
C20—C11—C12—C13	3.4 (4)	O6—C29—C30—C31	58.7 (3)
C1—C11—C12—C13	−177.9 (2)	C32—N2—C31—C30	−165.2 (3)
C20—C11—C12—O2	179.2 (2)	O7—C30—C31—N2	−52.6 (3)
C1—C11—C12—O2	−2.0 (4)	C29—C30—C31—N2	−177.2 (2)
P1—O2—C12—C11	73.7 (3)	C31—N2—C32—C33'	−57.1 (15)
P1—O2—C12—C13	−110.2 (2)	C31—N2—C32—C34	58.1 (17)
C11—C12—C13—C14	0.4 (4)	C31—N2—C32—C34'	69.9 (13)
O2—C12—C13—C14	−175.5 (3)	C31—N2—C32—C33	−66.8 (9)

### Hydrogen-bond geometry (Å, °)

**Table:**

*D*—H···*A*	*D*—H	H···*A*	*D*···*A*	*D*—H···*A*
O7—H7A···O3	0.82	1.87	2.683 (3)	172.
N2—H2B···O4^i^	0.90	1.95	2.746 (3)	147.
N2—H2A···O5^ii^	0.90	1.96	2.831 (3)	163.
N1—H1B···O5^i^	0.86	2.17	2.960 (3)	153.
N1—H1A···O4^iii^	0.86	2.07	2.907 (3)	164.

Symmetry codes: (i) *x*−1/2, −*y*+3/2, *z*; (ii) *x*−1/2, −*y*+3/2, *z*−1; (iii) *x*, *y*, *z*+1.
